# When migration leaves a clean trace: Decoupling migration from coalescence in the structured serial coalescent

**DOI:** 10.1101/2025.10.10.681523

**Published:** 2025-10-11

**Authors:** Hao Shen, John Novembre

**Affiliations:** 1 Department of Human Genetics, University of Chicago; 2 Department of Ecology and Evolution, University of Chicago

## Abstract

With the rapid accumulation of population genomic data across space and time, there is an urgent need for demographic inference methods that incorporate explicit time-series modeling, achieve fine spatial resolution, and ensure clear identifiability between migration and coalescence rates. To address this need, we investigate pairwise genealogical processes under the structured serial coalescent, deriving evolution equations for pairwise branch length distributions and related statistics. By classifying the resulting relationships according to their parameter dependencies and computational complexity, we identify a class that is not only computationally tractable but also determined solely by migration rates. Building on this theoretical basis, we propose an inference framework for fine-resolution, time-varying migration rates inference and demonstrate its feasibility through simulation. We further outline how this framework can be extended to the joint estimation of migration and coalescence rates.

## Introduction

Since Kingman introduced the coalescent (Kingman, 1982a,b), it has been widely used in population genetic theory and inferences. However, the standard coalescent theory typically oversimplifies reality by assuming contemporaneous sampling and panmixia, whereas empirical samples can come from different time periods and geographically structured populations with variable lineage migration and coalescence rates. These limitations have motivated key extensions: the serial coalescent for temporal stratification (Rodrigo and Felsenstein, 1999) and the structured coalescent for population subdivision (Notohara, 1990; Takahata, 1991). For models combining both temporal and spatial structure, terminology varies in the literature. Some authors retain ”structured coalescent” (Müller et al., 2017) while others use ”structured serial coalescent” (Ewing and Rodrigo, 2007). For clarity and distinction, we adopt the latter term throughout this paper.

These extensions of the standard coalescent have enabled powerful demographic inference tools, particularly for estimating coalescent effective population sizes (e.g., inverse of the instantaneous local coalescence rates) and migration rates.

For the serial coalescent, approaches using based on full gene genealogies using tools from Bayesian phylogentetics, were developed to estimate effective population sizes from temporally sampled data (Drummond et al., 2002, 2005). For the structured coalescent and structured serial coalescent, related methods using the full gene genealogy have been proposed to jointly infer migration rates and effective population sizes (Beerli and Felsenstein, 2001; Ewing et al., 2004; Volz, 2012; Vaughan et al., 2014; De Maio et al., 2015; Müller et al., 2017, 2018).

However, because these methods rely on information of the full genealogy, their computational scalability with respect to the number of lineages and demes remains limited—even with modern algorithmic advances. Consequently, demographic inference for systems with hundreds or thousands of demes remains impractical under these frameworks.

One effective way to solve the scalability problem is to use pairwise coalescence information instead of the whole genealogy. In the context of the structured coalescent, this means developing methods based on pairwise coalescence times. The distribution of pairwise coalescence times depends on both the migration rates and coalescence rates, while also being directly connected to empirical data through pairwise summary statistics. This makes pairwise coalescence times a useful basis for demographic inference.

For example, the methods EEMS (Petkova et al., 2016), FEEMS (Marcus et al., 2021), and FRAME (Shen and Novembre, 2025) all exploit the connection between expected pairwise coalescence times and the sample covariance matrix (McVean, 2009). Meanwhile, the method MAPS (Al-Asadi et al., 2019) uses the fact that the length distribution of long pairwise shared coalescent segments (LPSC segments, also know as the identity-by-descent or IBD tracts) is determined by the distribution of pairwise coalescence times (Palamara et al., 2012; Carmi et al., 2013). All of these methods are scalable to hundreds of demes. While earlier methods like EEMS, FEEMS, and MAPS relied heavily on the assumption of symmetric migration, work by (Lundgren and Ralph, 2019) and by (Shen and Novembre, 2025) with the FRAME method now enable fine-resolution inference for cases with asymmetric gene flow.

Despite these methodological advances, limitations persist. First, research across many domains requires time-series modeling to accommodate serial sampling and time-varying migration rates. In conservation genetics, samples collected at multiple time points can reveal how gene flow patterns change through time. In epidemiology, serially sampled viral genomes can be used to reconstruct transmission dynamics. In human evolutionary biology, ancient DNA provides genetic snapshots from different epochs, enabling reconstruction of migration histories within each period. FRAME, however, assumes migration–drift equilibrium under the structured coalescent and therefore cannot handle serial sampling or time-varying parameters. While it is possible to analyze data by dividing it into temporal slices and fitting each slice with an equilibrium model (as done demonstrated in the original MAPS and FRAME papers), it remains suboptimal compared to direct modeling under the structured serial coalescent framework. Second, these methods can have difficulty in distinguish the effects of migration rates and coalescence rates, leading to identifiability issues. (MAPS is an exception, but its effectiveness in asymmetric migration scenarios remains uncertain.)

These limitations motivate the need for a new demographic inference method that can: (1) preserve the scalability advantages of pairwise coalescence-based approaches, (2) directly incorporate serial sampling and time-varying parameters under the structured serial coalescent framework, and (3) effectively decouple the inference of migration rates from that of coalescence rates. In this paper we demonstrate that such a method is theoretically achievable.

Unlike the structured coalescent, where the equation for expected pairwise coalescence times in the structured coalescent was established (Strobeck, 1987) even before the theory’s formal development, the analogous theory for pairwise branch lengths in the structured serial coalescent - defined as the summed branch lengths from two sampled lineages to their most recent common ancestor (the serial sampling counterpart to pairwise coalescence times) - remains largely undeveloped. To bridge this theoretical gap and facilitate new inference methods, we develop the foundational theory for pairwise branch length dynamics under the structured serial coalescent framework.

Specifically, we derive evolution equations governing the probability density functions (PDFs) of pairwise branch lengths. By solving these equations, we uncover fundamental relationships among the PDFs and systematically classify them into distinct categories based on their parameter dependencies and computational complexity. This classification framework extends naturally to other quantities derived from pairwise branch lengths, as we demonstrate through parallel analysis of mean pairwise branch length dynamics and length distribution dynamics of LPSC segments.

The general applicability of this classification enables evaluation of each relationship class’s potential for demographic inference. Crucially, we identify a promising class that exhibits exclusive dependence on lineage migration rates with no influence from coalescence parameters, and computational tractability scaling as $O\left(d^{3}\right)$ where d represents the number of demes. These properties directly address the fundamental challenges identified earlier - they resolve the migration-coalescence identifiability problem while maintaining the scalability required for fine-resolution analysis.

This theoretical breakthrough establishes a foundation for two inference approaches. First, for studies focusing specifically on gene flow dynamics, these relationships enable highly scalable migration rate estimation that is decoupled from coalescence parameters. Second, joint estimation of migration and coalescence rates may also benefit from these relationships. A two-step approach—first estimating migration rates using these classes, then inferring coalescence rates conditioned on the migration parameters—could improve both identifiability and scalability, though the latter gain may be less pronounced than in migration-only inference. We outline inference frameworks for both approaches through a proof-of-concept example and demonstrate the feasibility of the first approach through preliminary simulation studies.

## The model

The structured serial coalescent process models the combined effects of backward migration between demes and coalescence events occurring when lineages reside in the same deme. We represent backward migration as a continuous-time jump process on a weighted directed graph with nodes {1,2,...,d}, each corresponding to a deme. The weight on the edge from node i to node j is denoted $m_{ij}(t)$, representing the backward migration rate from i to j at time t. Biologically, $m_{ij}(t)$ is the rate at which deme i receives ancestry from deme j. The transition rate matrix of this jump process is Q(t), and the associated Laplacian is defined as L(t)=-Q(t). The migration rates are encoded in the weighted adjacency matrix M(t)=Q(t)-diagQ(t). The coalescence rate in deme i is denoted by $\gamma_{i}(t)$, and all coalescence rates are collected into the vector $\gamma (t)=\left(\gamma_{i}(t)\right)$.

**Fig. 1.**
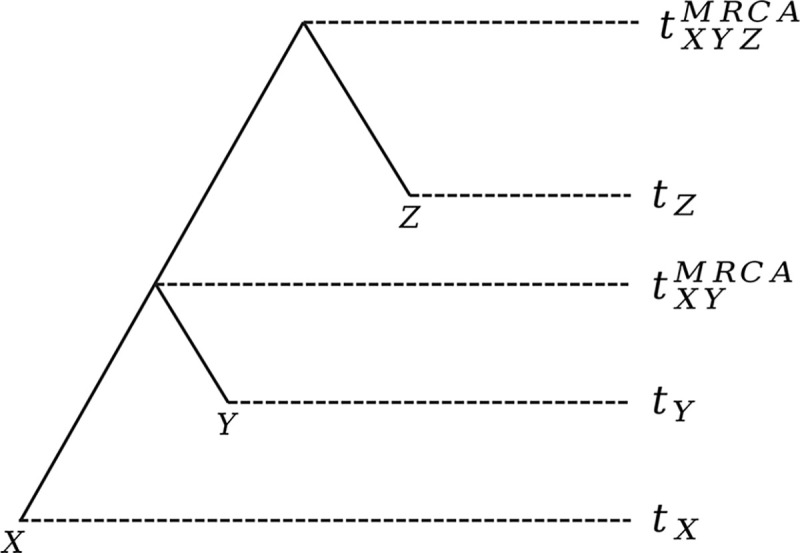
Pairwise branch length. A toy example showing how pairwise branch lengths are computed. Assume we have three samples X,Y, and Z sampled at times $t_{X},t_{Y}$, and $t_{Z}$, respectively. The pairwise branch length between X and Y is $\left(t_{XY}^{MRCA}-t_{X}\right)+\left(t_{XY}^{MRCA}-t_{Y}\right)$. Similarly, the pairwise branch length between X and Z is $\left(t_{XYZ}^{MRCA}-t_{X}\right)+\left(t_{XYZ}^{MRCA}-t_{Z}\right)$, and between Y and Z is $\left(t_{XYZ}^{MRCA}-t_{Y}\right)+\left(t_{XYZ}^{MRCA}-t_{Z}\right)$.

In our model, we study the pairwise genealogical process using the pairwise branch length, defined as the sum of the branch lengths of the two lineages up to their MRCA. This measure generalizes pairwise coalescence time by explicitly accounting for temporal offsets between samples and can be viewed as the distance of the two lineages in terms of branch length. For an illustration of how pairwise branch length is computed, see Figure 1.

Now let $B_{ij}^{x,y}$ denote the random variable representing the pairwise branch length between two lineages, one sampled from deme i at time x and the other from deme j at time y, where x≤y and time is measured backward from the present (t=0). Let $F_{ij}^{x,y}(b)=F_{ij}^{x,y,b}=P(B_{ij}^{x,y}\leq b)$ be the corresponding cumulative distribution function; $f_{ij}^{x,y}(b)=f_{ij}^{x,y,b}=\frac{\partial F_{ij}^{x,y,b}}{\partial b}$ be its probability density function; $f^{x,y}(b)=f^{x,y,b}=(f_{ij}^{x,y,b})$ be the matrix of the probability density functions (some times we will neglect b and just write $f^{x,y}$ to refer to the function). All the above functions have support in [y-x,+∞) and will be 0 for b<y-x.

In the next three sections, we will explore the relationships among the probability density functions as well as two related statistics (expected pairwise branch length and survival function of LPSC segment length).

### Relationships among probability density functions of pairwise branch length

**Fig. 2.**
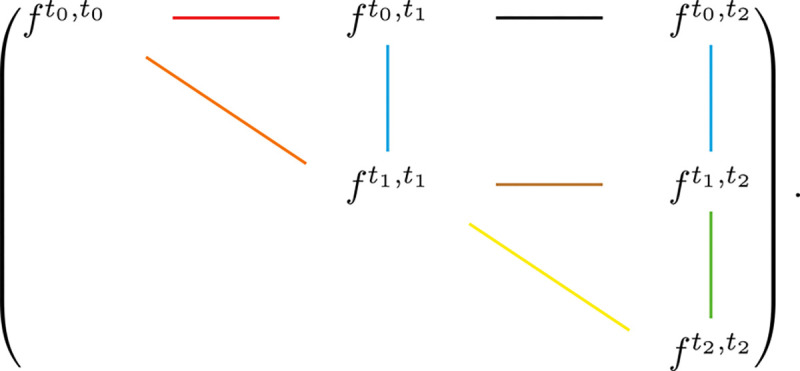
Graphical representation of the relationships among pairwise branch length probability density functions

We consider a toy example with three epochs: $\left[t_{0},t_{1}\right),\left[t_{1},t_{2}\right)$, and $\left[t_{2},\infty \right)$, which we label as epoch 0, epoch 1, and epoch 2, respectively. The migration and coalescence rates are assumed to be piecewise constant in the first two epoches, that is, $L(x)=L^{0},\gamma (x)=\gamma^{0}$ for $x\in \left[t_{0},t_{1}\right)$ and $L(x)=L^{1},\gamma (x)=\gamma^{1}$ for $x\in \left[t_{1},t_{2}\right)$ (The migration and coalescence rates in the last epoch are not specified, as they are not used in this section). We now investigate relationships among the following time-ordered pairs of pairwise branch length pdf matrices: $\left(f^{t_{0},t_{1}},f^{t_{1},t_{1}}\right)$;$\left(f^{t_{0},t_{2}},f^{t_{1},t_{2}}\right)$;$\left(f^{t_{1},t_{2}},f^{t_{2},t_{2}}\right)$;$\left(f^{t_{0},t_{0}},f^{t_{1},t_{1}}\right)$;$\left(f^{t_{1},t_{1}},f^{t_{2},t_{2}}\right)$;$\left(f^{t_{0},t_{0}},f^{t_{0},t_{1}}\right)$;$\left(f^{t_{1},t_{1}},f^{t_{1},t_{2}}\right)$; $\left(f^{t_{0},t_{1}},f^{t_{0},t_{2}}\right)$. By abuse of notation, we will also use these pairs to denote the corresponding relationships. These 8 relationships correspond to the 8 edges in Fig. 2.

Graphically, these 8 edges in Fig. 2 fall naturally into three categories: the vertical edges, the diagonal edges and the horizontal edges. In what follows, we show that this graphical classification corresponds to a classification of the relationships themselves in terms of representational and computational complexity.

We first examine the relationship ($f^{t_{0},t_{1}},f^{t_{1},t_{1}}$), which is represented by a vertical edge. To this end, we study the evolution of $f^{x,t_{1},b}$ for $x\in \left[t_{0},t_{1}\right)$. As we move from x to x+∆t, coalescence does not occur, and only migration of the first lineage is relevant. This yields the following equation

(1) $$f_{ij}^{x,t_{1},b}=\sum_{k\neq i}m_{ik}\left(x\right)\Delta tf_{kj}^{x+\Delta t,t_{1},b-\Delta t}+\left(1-\sum_{k\neq i}m_{ik}\left(x\right)\Delta t\right)f_{ij}^{x+\Delta t,t_{1},b-\Delta t}.$$

Expanding $f_{\alpha j}^{x+\Delta t,t_{1},b-\Delta t}$ as $f_{\alpha j}^{x,t_{1},b}+\frac{\partial f_{\alpha j}^{x,t_{1},b}}{\partial x}\Delta t-\frac{\partial f_{\alpha j}^{x,t_{1},b}}{\partial b}\Delta t$ and ignoring higher-order terms, we cancel Δt on both sides and obtain

(2) $$\frac{\partial f_{ij}^{x,t_{1},b}}{\partial x}-\frac{\partial f_{ij}^{x,t_{1},b}}{\partial b}=\left(\sum_{k\neq i}m_{ik}(x)\right)f_{ij}^{x,t_{1},b}-\sum_{k\neq i}m_{ik}(x)f_{kj}^{x,t_{1},b}.$$

In matrix form, this partial differential equation becomes

(3) $$\frac{\partial f^{x,t_{1},b}}{\partial x}-\frac{\partial f^{x,t_{1},b}}{\partial b}=L(x)f^{x,t_{1},b}=L^{0}f^{x,t_{1},b}.$$

with the boundary condition of known $f^{t_{1},t_{1},b}$. This PDE has the explicit solution

(4) $$f^{x,t_{1},b}=e^{-L^{0}\left(t_{1}-x\right)}f^{t_{1},t_{1},b-\left(t_{1}-x\right)}.$$

Taking the limit as $x\to t_{0}$, we obtain the relationship ($f^{t_{0},t_{1}},f^{t_{1},t_{1}}$). Following similar logic, the relationships ($f^{t_{0},t_{2}},f^{t_{1},t_{2}}$) and ($f^{t_{1},t_{2}},f^{t_{2},t_{2}}$), which are likewise represented by vertical edges, can also be computed. These three relationships can be jointly expressed as

(5a) $$f^{t_{0},t_{1},b}=e^{-L^{0}\left(t_{1}-t_{0}\right)}f^{t_{1},t_{1},b-\left(t_{1}-t_{0}\right)},$$

(5b) $$f^{t_{0},t_{2},b}=e^{-L^{0}\left(t_{1}-t_{0}\right)}f^{t_{1},t_{2},b-\left(t_{1}-t_{0}\right)},$$

(5c) $$f^{t_{1},t_{2},b}=e^{-L^{1}\left(t_{2}-t_{1}\right)}f^{t_{2},t_{2},b-\left(t_{2}-t_{1}\right)}.$$

These relationships are particularly valuable for two reasons. First, they depend only on backward migration rates $\left(f^{t_{0},t_{1}},f^{t_{1},t_{1}}\right)$ and ($f^{t_{0},t_{2}},f^{t_{1},t_{2}}$) depend on $L^{0}$ (we represent the corresponding edges in blue in Fig. 2), while ($f^{t_{1},t_{2}},f^{t_{2},t_{2}}$) depends on $L^{1}$. This makes them a promising tool for disentangling migration dynamics from coalescence effects (or effective population sizes) in complex migration–drift scenarios. Second, the computational complexity of these relationships is at most $O\left(d^{3}\right)$, and can often be reduced further by exploiting the (potentially) sparse structure of the Laplacian matrices. This scalability makes it possible to perform gene flow or migration inference at an unprecedented fine resolution, involving hundreds or even thousands of demes. As we shall see, the class of relationships represented by the vertical edges is the only one that possesses these favorable properties, and should therefore be fully exploited to facilitate inference.

Now we turn to the relationships ($f^{t_{0},t_{0}},f^{t_{1},t_{1}}$), which corresponds to a diagonal edge in Figure.2. Let $f^{x,b}=f^{x,x,b}$ denote the density matrix when the two lineages are sampled at the same time. For $x\in \left[t_{0},t_{1}\right)$, the associated partial differential equation is

(6) $$\frac{\partial f^{x,b}}{\partial x}-2\frac{\partial f^{x,b}}{\partial b}=diag\left\{\gamma^{0}\right\}\;diag\{f^{x,b}\}+L^{0}f^{x,b}+f^{x,b}{\left(L^{0}\right)}^{T},$$

with boundary condition of known $f^{t_{1},b}$ and $f^{x,0}=diag\left\{\gamma^{0}\right\}$ for $x\in \left[t_{0},t_{1}\right)$. This equation is derived in a manner similar to equation (3), with the key difference being that both lineages can migrate independently, and coalescence may occur when they are in the same deme. The PDE remains linear once we vectorize both sides.

Let $E_{i}$ denote the matrix with a 1 in the (i,i)-th entry and zeros elsewhere, and let $\epsilon_{i}=vec\left(E_{i}\right)$ be its vectorization. Define $S^{0}=L^{0}⊗I+I⊗L^{0}+\sum_{i=1}^{d}\gamma_{i}^{0}\epsilon_{i}\epsilon_{i}^{T}$, where ⊗ denotes the Kronecker product, define and $G^{0}=\sum_{i=1}^{d}\gamma_{i}^{0}\epsilon_{i}$. Then the solution to equation (6) is given by

(7) $$vec(f^{x,b})=\left\{\begin{array}{ll} e^{-\frac{1}{2}S^{0}b}G^{0} & b\in \left[0,\;2\left(t_{1}-x\right)\right)\\ e^{-S^{0}\left(t_{1}-x\right)}\;vec(f^{t_{1},b-2\left(t_{1}-x\right)}) & b\in \left[2\left(t_{1}-x\right),+\infty \right) \end{array}\right.$$

Taking the limit as $x\to t_{0}$, we obtain the relationship ($f^{t_{0},t_{0}},f^{t_{1},t_{1}}$). A similar analysis applies to the relationship ($f^{t_{1},t_{1}},f^{t_{2},t_{2}}$), which is represented by another diagonal edge. Together, the relationships represented by the diagonal edges can be written jointly as

(8a) $$vec(f^{t_{0},b})=\left\{\begin{array}{ll} e^{-\frac{1}{2}S^{0}b}G^{0} & b\in \left[0,\;2\left(t_{1}-t_{0}\right)\right)\\ e^{-S^{0}\left(t_{1}-t_{0}\right)}\;vec(f^{t_{1},b-2\left(t_{1}-t_{0}\right)}) & b\in \left[2\left(t_{1}-t_{0}\right),+\infty \right) \end{array}\right.$$

(8b) $$vec(f^{t_{1},b})=\left\{\begin{array}{ll} e^{-\frac{1}{2}S^{1}b}G^{1} & b\in \left[0,\;2\left(t_{2}-t_{1}\right)\right)\\ e^{-S^{1}\left(t_{2}-t_{1}\right)}\;vec(f^{t_{2},b-2\left(t_{2}-t_{1}\right)}) & b\in \left[2\left(t_{2}-t_{1}\right)+\infty \right) \end{array}\right.$$

where $S^{1}=L^{1}⊗I+I⊗L^{1}+\sum_{i=1}^{d}\gamma_{i}^{1}\epsilon_{i}\epsilon_{i}^{T},G^{1}=\sum_{i=1}^{d}\gamma_{i}^{1}\epsilon_{i}$.

Before turning to the relationships represented by the horizontal edges, we would like to note two points. First, when the two lineages evolve together in $f^{x,x,b}$, the model simplifies to a structured coalescent model. The probability density function of the pairwise coalescence times can be derived using the fact that the pairwise branch lengths are twice the pairwise coalescence times in the structured coalescent. Second, as $t_{2}$ goes to infinity in equation (8b, we obtain the stationary distribution of pairwise branch length under a structured coalescent model with migration rates coded by $L^{1}$ and coalescence rates coded by $\gamma^{1}$. The stationary distribution of pairwise branch length distribution satisfies

(9) $$vec(f^{1*,b})=e^{-\frac{1}{2}S^{1}b}G^{1},$$

where $f^{1*,b}=\underset{t_{2}\to +\infty }{lim}f^{t_{1},b}$.

Because $S^{0}$ and $S^{1}$ depend on both migration and coalescence rates, the relationships represented by the diagonal edges are generally more complex and computationally intensive than those represented by the vertical edges. However, as we shall see next, they remain relatively straightforward to derive and interpret compared with the relationships represented by the horizontal edges, and thus serve as useful candidates for inferring coalescence rates or effective population sizes.

For the relationships represented by the horizontal edges, namely ($f^{t_{0},t_{0}},f^{t_{0},t_{1}}$), ($f^{t_{1},t_{1}},f^{t_{1},t_{2}}$), and ($f^{t_{0},t_{1}},f^{t_{0},t_{2}}$), it is not easy to directly formulate and solve the corresponding PDEs. Since we have already established the relationships corresponding to the vertical and diagonal edges, we derive those for the horizontal edges indirectly (for details of the derivation, see Supplementary Information A), which yields

(10a) $$vec(f^{t_{0},t_{0},b})=\left\{\begin{array}{ll} e^{-\frac{1}{2}S^{0}b}G^{0} & b\in \left[0,\;2\left(t_{1}-t_{0}\right)\right)\\ e^{-S^{0}\left(t_{1}-t_{0}\right)}\;vec(e^{L^{0}\left(t_{1}-t_{0}\right)}f^{t_{0},t_{1},b-\left(t_{1}-t_{0}\right)}) & b\in \left[2\left(t_{1}-t_{0}\right),+\infty \right) \end{array}\right.$$

(10b) $$vec(f^{t_{1},t_{1},b})=\left\{\begin{array}{ll} e^{-\frac{1}{2}S^{1}b}G^{1} & b\in \left[0,\;2\left(t_{2}-t_{1}\right)\right)\\ e^{-S^{1}\left(t_{2}-t_{1}\right)}\;vec(e^{L^{1}\left(t_{2}-t_{1}\right)}f^{t_{1},t_{2},b-\left(t_{2}-t_{1}\right)}) & b\in \left[2\left(t_{2}-t_{1}\right),+\infty \right) \end{array}\right.$$

(10c) $$vec(e^{L^{0}(t_{1}-t_{0})}f^{t_{0},t_{1},b})\;=\left\{\begin{array}{ll} e^{-\frac{1}{2}S^{1}\left[b-\left(t_{1}-t_{0}\right)\right]}G^{1} & b\in \left[t_{1}-t_{0},2t_{2}-t_{1}-t_{0}\right)\\ e^{-S^{1}\left(t_{2}-t_{1}\right)}\;vec(e^{L^{1}\left(t_{2}-t_{1}\right)}e^{L^{0}\left(t_{1}-t_{0}\right)}f^{t_{0},t_{2},b-\left(t_{2}-t_{1}\right)}) & b\in \left[2t_{2}-t_{1}-t_{0},+\infty \right) \end{array}\right.$$

These equations are highly complex both in terms of representation and computation. The difficulty arises from the fact that when we trace lineage 2 in $f^{x,y,b}$ along y, the process is not purely migrational: we must also account for the location of lineage 1 at the same time point. If lineage 1 happens to be in the same deme as lineage 2, there is a positive probability that they coalesce. Moreover, for lineage 1 to evolve from time x to time y, it must pass through all changes in the migration rates during the interval [x,y]. This is why the relationship ($f^{t_{0},t_{1}},f^{t_{0},t_{2}}$), represented by the black horizontal edge in Fig. 2, is particularly intricate, as it depends simultaneously on $L^{0},L^{1}$, and $\gamma^{1}$.

Since all relationships represented by the horizontal edges can be effectively derived from those represented by the vertical and diagonal edges, and are typically much more complicated than the other two categories, it is important to avoid using them for inference. In a demographic inference framework based on pairwise branch lengths, one should rely only on relationships represented by vertical edges when estimating backward migration rates, and use both vertical and diagonal edges for the joint inference of migration rates and coalescence rates.

### Relationships among expected pairwise branch lengths

The relationships among the probability density functions (pdfs) of pairwise branch lengths naturally extend to summary statistics derived from them. For example, consider the expected pairwise branch length $\bar{B^{x,y}}$. In the structured coalescent framework, the analogous quantity - expected pairwise coalescence times - are directly related to sample covariance structure (McVean, 2009), a connection exploited by methods including EEMS (Petkova et al., 2016), FEEMS (Marcus et al., 2021), and FRAME (Shen and Novembre, 2025). As we demonstrate in Supplementary Information D, such connection can extend to serially sampled data with slight modifications. Furthermore, advances in tree sequence reconstruction now allow for the direct estimation of $\bar{B^{x,y}}$ from inferred branch lengths, though this requires careful consideration of the demographic assumptions inherent in the reconstruction process. Here, we establish the relationships among expected pairwise branch lengths in the structured serial coalescent.

**Fig. 3.**
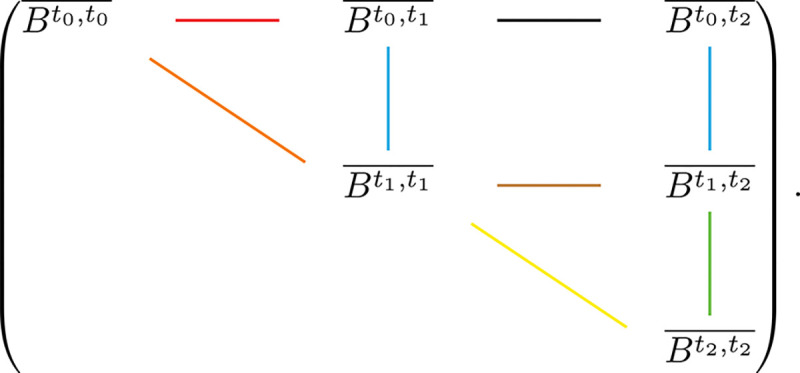
Graphical representation of the relationships among expected pairwise branch lengths

Similar to last section, we study a three-epoch toy model. The 8 relationships we would like to study are $\left(\bar{B^{t_{0},t_{1}}},\bar{B^{t_{1},t_{1}}}\right)$; $\left(\bar{B^{t_{0},t_{2}}},\bar{B^{t_{1},t_{2}}}\right)$; $\left(\bar{B^{t_{1},t_{2}}},\bar{B^{t_{2},t_{2}}}\right)$; $\left(\bar{B^{t_{0},t_{0}}},\bar{B^{t_{1},t_{1}}}\right)$; $\left(\bar{B^{t_{1},t_{1}}},\bar{B^{t_{2},t_{2}}}\right)$; $\left(\bar{B^{t_{0},t_{0}}},\bar{B^{t_{0},t_{1}}}\right)$; $\left(\bar{B^{t_{1},t_{1}}},\bar{B^{t_{1},t_{2}}}\right)$; $\left(\bar{B^{t_{0},t_{1}}},\bar{B^{t_{0},t_{2}}}\right)$. These relationships corresponds to edges in Fig. 3.

We now study $\left(\bar{B^{t_{0},t_{1}}},\bar{B^{t_{1},t_{1}}}\right)$. When $x\in \left[t_{0},t_{1}\right)$, this expectation satisfies the partial differential equation

(11) $$\frac{\partial \bar{B^{x,t_{1}}}}{\partial x}=L^{0}\bar{B^{x,t_{1}}}-1_{d\times d},$$

with boundary condition of known $\bar{B^{t_{1},t_{1}}}$. Using the identities $L1_{d\times d}=0$ and $e^{L^{0}\alpha }1_{d\times d}=1_{d\times d}$ for any α, the solution is given by

(12) $$\bar{B^{x,t_{1}}}=e^{-L^{0}\left(t_{1}-x\right)}\bar{B^{t_{1},t_{1}}}+\left(t_{1}-x\right)1_{d\times d}.$$

Taking the limit as $x\to t_{0}$, we get the relationship $\left(\bar{B^{t_{0},t_{1}}},\bar{B^{t_{1},t_{1}}}\right)$. Following the same logic, the relationships represented by other vertical edges can also be computed. These relationships can be jointly written as

(13a) $$\bar{B^{t_{0},t_{1}}}=e^{-L^{0}\left(t_{1}-t_{0}\right)}\bar{B^{t_{1},t_{1}}}+\left(t_{1}-t_{0}\right)1_{d\times d},$$

(13b) $$\bar{B^{t_{0},t_{2}}}=e^{-L^{0}\left(t_{1}-t_{0}\right)}\bar{B^{t_{1},t_{2}}}+\left(t_{1}-t_{0}\right)1_{d\times d},$$

(13c) $$\bar{B^{t_{1},t_{2}}}=e^{-L^{1}\left(t_{2}-t_{1}\right)}\bar{B^{t_{2},t_{2}}}+\left(t_{2}-t_{1}\right)1_{d\times d}.$$

The relationships corresponding to the diagonal edges can also be derived directly. Let $\bar{B^{x}}=\bar{B^{x,x}}=\int_{0}^{+\infty }bf^{x,b}db$ denote the matrix of expected pairwise branch lengths when both lineages are sampled at time x. For $x\in \left[t_{0},t_{1}\right)$, this satisfies the ordinary differential equation

(14) $$\frac{d\bar{B^{x}}}{dx}=diag\left\{\gamma \right\}\;diag\{\bar{B^{x}}\}+L^{0}\bar{B^{x}}+\bar{B^{x}}{\left(L^{0}\right)}^{T}-2_{d\times d}.$$

Vectorizing and solving this equation gives

(15) $$vec(\bar{B^{x}}-\bar{B^{0*}})=e^{-S^{0}\left(t_{1}-x\right)}\;vec(\bar{B^{t_{1}}}-\bar{B^{0*}}),$$

where $\bar{B^{0*}}$ is the equilibrium expected pairwise branch length under the parameter set ($L^{0},\gamma^{0}$) and satisfies the equation

(16) $$diag\left\{\gamma^{0}\right\}\;diag\{\bar{B^{0*}}\}+L^{0}\bar{B^{0*}}+\bar{B^{0*}}{\left(L^{0}\right)}^{T}=2_{d\times d}.$$

Now letting $x\to t_{0}$ and applying the same logic to the epoch $\left[t_{1},t_{2}\right)$, we obtain the joint equation for the relationships represented by the diagonal edges

(17a) $$vec(\bar{B^{t_{0}}}-\bar{B^{0*}})=e^{-S^{0}\left(t_{1}-t_{0}\right)}vec(\bar{B^{t_{1}}}-\bar{B^{0*}}),$$

(17b) $$vec(\bar{B^{t_{1}}}-\bar{B^{1*}})=e^{-S^{1}\left(t_{2}-t_{1}\right)}vec(\bar{B^{t_{2}}}-\bar{B^{1*}}),$$

where $\bar{B^{1*}}$ satisfies

(18) $$diag\left\{\gamma^{1}\right\}\;diag\{\bar{B^{1*}}\}+L^{1}\bar{B^{1*}}+\bar{B^{1*}}{\left(L^{1}\right)}^{T}=2_{d\times d}.$$

The relationships corresponding to the horizontal edges can also be derived indirectly (for a detailed derivation, see Supplementary Information B), and can be written as

(19a) $$vec(\bar{B^{t_{0},t_{0}}}-\bar{B^{0*}})=e^{-S^{0}\left(t_{1}-t_{0}\right)}\;vec(e^{L^{0}\left(t_{1}-t_{0}\right)}\bar{B^{t_{0},t_{1}}}-\left(t_{1}-t_{0}\right)1_{d\times d}-\bar{B^{0*}}),$$

19b) $$vec(\bar{B^{t_{1},t_{1}}}-\bar{B^{1*}})=e^{-S^{1}\left(t_{2}-t_{1}\right)}\;vec(e^{L^{1}\left(t_{2}-t_{1}\right)}\bar{B^{t_{0},t_{1}}}-\left(t_{2}-t_{1}\right)1_{d\times d}-\bar{B^{1*}}),$$

(19c) $$vec(e^{L^{0}\left(t_{1}-t_{0}\right)}\bar{B^{t_{0},t_{1}}}-\left(t_{1}-t_{0}\right)1_{d\times d}-\bar{B^{1*}})\;=e^{-S^{1}\left(t_{2}-t_{1}\right)}\;vec(e^{L^{1}\left(t_{2}-t_{1}\right)}e^{L^{0}\left(t_{1}-t_{0}\right)}\bar{B^{t_{0},t_{2}}}-\left(t_{2}-t_{0}\right)1_{d\times d}-\bar{B^{1*}}).$$

As expected, the relationships corresponding to the vertical edges depend only on migration rates and are relatively easy to compute, whereas those corresponding to the horizontal edges are the most complex in terms of both representation and computation.

### Relationships among survival functions of LPSC segment lengths

In classical coalescent as well structured coalescent, it is well known that the length distribution of long pairwise shared coalescence segments (LPSC segments, or identity-by-descent tracts) is determined by the distribution of pairwise coalescence time (Palamara et al., 2012; Carmi et al., 2013) and can thus be used for demographic inference. This is the idea underlying MAPS (Al-Asadi et al., 2019), which infers the migration surfaces and coalescence rates based on the LPSC length distribution. In structured serial coalescent, following the logic of the previous two sections, we establish the relationships among the survival functions of the LPSC segment lengths.

Let $\rho^{x,y,\mu }$ be the matrix of survival functions such that it’s $(i,j{)}_{\text{th}}$ element is the probability that a LPSC segment between two randomly sampled lineages from deme i at time x and deme j at time y has length larger than μ. The probability of a random LPSC segment has length larger than μ given the pairwise branch length b and recombination rate r is simply $e^{-rbu}$. And we obtain

(20) $$\rho^{x,y,\mu }=\int_{0}^{+\infty }e^{-rb\mu }f^{x,y,b}db.$$

**Fig. 4.**
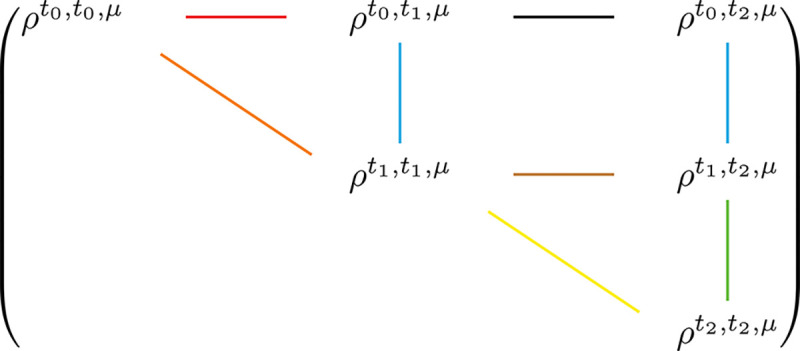
Graphical representation of the relationships among survival functions of LPSC segment lengths

Here again we employ the three epoch toy model, and we would like to investigate the 8 relationships $\left(\rho^{t_{0},t_{1},\mu },\rho^{t_{1},t_{1},\mu }\right)$; $\left(\rho^{t_{0},t_{2},\mu },\rho^{t_{1},t_{2},\mu }\right)$; $\left(\rho^{t_{1},t_{2},\mu },\rho^{t_{2},t_{2},\mu }\right)$; $\left(\rho^{t_{0},t_{0},\mu },\rho^{t_{1},t_{1},\mu }\right)$; $\left(\rho^{t_{1},t_{1},\mu },\rho^{t_{2},t_{2},\mu }\right)$; $\left(\rho^{t_{0},t_{0},\mu },\rho^{t_{0},t_{1},\mu }\right)$; ($\rho^{t_{1},t_{1},\mu },\rho^{t_{1},t_{2},\mu }$); ($\rho^{t_{0},t_{1},\mu },\rho^{t_{0},t_{2},\mu }$). Again, these 8 relationships correspond to 8 edges in Fig. 4.

The easiest way to study the relationships represented by the vertical edges here to directly write out the integration out and use the relationships among the probability density functions, instead of solving the PDEs. Writing $L^{0}+r\mu I,L^{1}+r\mu I$ as $L_{\mu }^{0},L_{\mu }^{1}$ and using equation (5a), (5b) and (5c) yields

(21a) $$\rho^{t_{0},t_{1},\mu }=e^{-L_{\mu }^{0}\left(t_{1}-t_{0}\right)}\rho^{t_{1},t_{1},\mu },$$

(21b) $$\rho^{t_{0},t_{2},\mu }=e^{-L_{\mu }^{0}\left(t_{1}-t_{0}\right)}\rho^{t_{1},t_{2},\mu },$$

(21c) $$\rho^{t_{1},t_{2},\mu }=e^{-L_{\mu }^{1}\left(t_{2}-t_{1}\right)}\rho^{t_{2},t_{2},\mu }.$$

For the relationships represented by the diagonal edges, we can also derive analogous expressions. Applying equation (8a), (8b), with $\rho^{x,x,\mu }=\rho^{x,\mu }$, $S^{0}+2r\mu I=S_{\mu }^{0},S^{1}+2r\mu I=S_{\mu }^{1}$, we obtain

(22a) $$vec\left(\rho^{t_{0},\mu }\right)=2[I-e^{-S_{\mu }^{0}\left(t_{1}-t_{0}\right)}]{\left(S_{\mu }^{0}\right)}^{-1}G^{0}+e^{-S_{\mu }^{0}\left(t_{1}-t_{0}\right)}\;vec\left(\rho^{t_{1},\mu }\right),$$

(22b) $$vec\left(\rho^{t_{1},\mu }\right)=2[I-e^{-S_{\mu }^{1}\left(t_{2}-t_{1}\right)}]{\left(S_{\mu }^{1}\right)}^{-1}G^{1}+e^{-S_{\mu }^{1}\left(t_{2}-t_{1}\right)}\;vec\left(\rho^{t_{2},\mu }\right).$$

Finally the relationships corresponding to the horizontal edges can also be derived indirectly, which are given by

(23a) $$vec\left(\rho^{t_{0},t_{0},\mu }\right)=2[I-e^{-S_{\mu }^{0}\left(t_{1}-t_{0}\right)}]{\left(S_{\mu }^{0}\right)}^{-1}G^{0}+e^{-S_{\mu }^{0}\left(t_{1}-t_{0}\right)}\;vec(e^{L_{\mu }^{0}\left(t_{1}-t_{0}\right)}\rho^{t_{0},t_{1},\mu }),$$

(23b) $$vec\left(\rho^{t_{1},t_{1},\mu }\right)=2[I-e^{-S_{\mu }^{1}\left(t_{2}-t_{1}\right)}]{\left(S_{\mu }^{1}\right)}^{-1}G^{1}+e^{-S_{\mu }^{1}\left(t_{2}-t_{1}\right)}\;vec(e^{L_{\mu }^{1}\left(t_{2}-t_{1}\right)}\rho^{t_{1},t_{2},\mu }),$$

(23c) $$e^{L_{\mu }^{0}\left(t_{1}-t_{0}\right)}\;vec\left(\rho^{t_{0},t_{1},\mu }\right)=2[I-e^{-S_{\mu }^{1}\left(t_{2}-t_{1}\right)}]{\left(S_{\mu }^{1}\right)}^{-1}G^{1}+e^{-S_{\mu }^{1}\left(t_{2}-t_{1}\right)}\;vec(e^{L_{\mu }^{1}\left(t_{2}-t_{1}\right)}e^{L_{\mu }^{0}\left(t_{1}-t_{0}\right)}\rho^{t_{0},t_{2},\mu }).$$

A step-by-step derivation of the equations presented in this section can be found in the Supplementary Information C. Once again, we observe that the relationships corresponding to the vertical edges are the most well-behaved, while those corresponding to the horizontal edges are the most complex in terms of both representation and computation.

### Inference framework

Here we study how the above relationships can be used to establish inference frameworks. Similar to the previous sections, we set up a proof-of-concept example by assuming that samples are available at three time points, $t_{0},t_{1}$, and $t_{2}$, and that the goal is to infer the ’effective’ demographic parameters in $\left[t_{0},t_{1}\right)$ and $\left[t_{1},t_{2}\right)$ using the relationships among the expected pairwise coalescence times, i.e., the $\bar{B^{t_{i},t_{j}}}$ matrices.

Although the $\bar{B^{t_{i},t_{j}}}$ matrices are not directly observable, they are naturally connected to sample variance structures and reconstructed tree sequences, as discussed in previous sections. The protocols below do not detail the establishment of these connections but instead present a higher-level inference framework. For convenience, we refer to sample variance structures and reconstructed tree sequences collectively as ’corresponding data’, and on this basis propose two demographic inference approaches: one specialized for inferring migration rates, and another designed for the joint estimation of migration and coalescence rates.

### Pure migration rates inference

For pure migration rates inference in the proof-of concept example, we follow a simple three-step protocol:

1. Estimate the $\bar{B^{t_{2},t_{2}}}$ matrix from the corresponding data. This estimation can be straightforward if genetic samples are available from every deme. However, for cases involving vacant demes (those without samples), imputation becomes necessary. One strategy is to use spatial imputation, which leverages geographical proximity to infer values for the vacant demes based on the directly estimated submatrix of $\bar{B^{t_{2},t_{2}}}$ that represents the demes with samples. Alternatively, a model-based imputation approach can be employed. This method operates under the assumption that the stochastic process governing lineages before time $t_{2}$ was driven by a set of equilibrium migration rates ($L^{\infty }$) and coalescence rates ($\gamma^{\infty }$). In other words, it assumes $\bar{B^{t_{2},t_{2}}}=\bar{B^{\infty }}$, where the equilibrium matrix is defined by the equation:
(24) $$diag{\;\gamma }^{\infty }\;diag\bar{B^{\infty }}+L^{\infty }\bar{B^{\infty }}+\bar{B^{\infty }}{\left(L^{\infty }\right)}^{T}=2_{d\times d}.$$
  Under this assumption, established equilibrium-based methods, such as FRAME (Shen and Novembre, 2025), can be used to first infer the parameters $L^{\infty }$ and $\gamma^{\infty }$, which in turn provides an estimate for the complete $\bar{B^{t_{2},t_{2}}}$ matrix.
2. Estimate $\bar{B^{t_{1},t_{2}}}$ and $L^{1}$ based on corresponding data and estimated $\bar{B^{t_{2},t_{2}}}$ using equation (13c). From equation (13c), we know $\bar{B^{t_{1},t_{2}}}$ is a function of $\bar{B^{t_{2},t_{2}}}$ and $L^{1}$, since in the first step we already have the inferred $\bar{B^{t_{2},t_{2}}}$, we can write $\bar{B^{t_{1},t_{2}}}$ solely as a function of $L^{1}$. Consequently, a connection between $L^{1}$ and the corresponding data is mediated through $\bar{B^{t_{1},t_{2}}}$. This established relationship allows for the estimation of $L^{1}$. Notably, if vacant demes prevent the direct estimation of $\bar{B^{t_{1},t_{2}}}$ from corresponding data, a model based imputation step can be performed simultaneously with the estimation of $L^{1}$.
3. Estimate $\bar{B^{t_{0},t_{2}}}$, and $L^{0}$ based on corresponding data and estimated $\bar{B^{t_{1},t_{2}}}$ using equation (5b). Here the logic is similar to that in step 2. One thing to be mentioned here is that equation (5a) can also be used to help estimate $L^{0}$ if $\bar{B^{t_{1},t_{2}}}$ can be properly estimated from corresponding data. In practice people can either use a more convenient one or use both of them.

### Joint inference of migration rates and coalescence rates

While the joint inference of migration and coalescence rates involves additional steps, the procedure for estimating migration rates remains consistent with the gene-flow-only protocol. Therefore, for these established steps, we will simply repeat the instructions without further explanation. New procedures specific to joint inference will be explained in detail. The protocol in the proof-of-concept example is as follows:

1. Estimate the $\bar{B^{t_{2},t_{2}}}$ matrix from the corresponding data.
2. Estimate $\bar{B^{t_{1},t_{2}}}$ and $L^{1}$ based on corresponding data and estimated $\bar{B^{t_{2},t_{2}}}$ using equation (13c).
3. Estimate $\bar{B^{t_{1},t_{1}}}$ and $\gamma^{1}$ based on corresponding data, estimated $\bar{B^{t_{2},t_{2}}}$ and estimated $L^{1}$ using equation (8b). The idea here again is to use $\bar{B^{t_{1},t_{1}}}$ as a mediater to connect $\gamma^{1}$ to corresponding data. An important thing to mention is that we directly use the estimate of $L^{1}$ from last step instead of estimating it jointly with $L^{1}$.
4. Estimate $\bar{B^{t_{0},t_{1}}},\bar{B^{t_{0},t_{2}}},L^{0}$ based on corresponding data, estimated $\bar{B^{t_{1},t_{1}}}$ and estimated $\bar{B^{t_{1},t_{2}}}$ using equation (5a) and (5b). The underlying logic is analogous to step 3 of the gene-flow inference protocol. The key distinction is that $\bar{B^{t_{1},t_{1}}}$ is now already estimated from the previous step, which simplifies the direct application of equation (5a). Again, in practice people can either use a more convenient equation or use both of them.
5. Estimate $\bar{B^{t_{0},t_{0}}}$ and $\gamma^{0}$ based corresponding data and estimated $\bar{B^{t_{1},t_{1}}}$ using equation (8a). This step follows the same logic as step 3.

While the proof-of-concept example demonstrates the core framework, it can be generalized to cases with more than two time slices and other data types, such as sample covariance structures or LPSC segment data.

### Simulation

Here, we test the feasibility of our first inference approach—inferring pure migration rates—through simulation studies. Similar to the previous analysis, we consider a model with three epochs, $\left[t_{0},t_{1}\right),\left[t_{1},t_{2}\right)$, and $\left[t_{2},t_{3}\right)$. In each epoch, we impose a different migration topology and assign random effective population sizes (i.e., coalescence rates). For each combination of topologies, we sample 10 haplotype lineages per deme per epoch and use msprime (Baumdicker et al., 2022) to simulate 10*,*000 tree sequences. Parameters are then inferred from the pairwise branch length data of these simulated datasets as follows:

1. Estimate the $\bar{B^{t_{2},t_{2}}}$ matrix from the simulated dataset, which we denote by $\hat{B^{t_{2},t_{2}}}$. For simplicity, in this step we directly use the sample mean of pairwise branch length, i.e. $\hat{B_{\text{sample}}^{t_{2},t_{2}}}$, since we have samples from all the demes.
2. Estimate $\bar{B^{t_{1},t_{2}}}$ and $L^{1}$ based on corresponding data and estimated $\bar{B^{t_{2},t_{2}}}$ using equation (13c). From the last step, we already have $\hat{B^{t_{2},t_{2}}}$, and we first obtain a preliminary estimate of $\bar{B^{t_{1},t_{2}}}$ by taking the sample mean of the pairwise branch lengths, denoted $\hat{B_{\text{sample}}^{1,t_{2}}}$. We then infer $L^{1}$ by solving an optimization problem with objective ${\left\|\varepsilon_{r}^{1}\right\|}^{2}+\lambda \Psi$, where $\varepsilon_{r}^{1}$ is the relative error matrix of equation (13c) computed from $\hat{B^{t_{2},t_{2}}}$ and $\hat{B_{\text{sample}}^{t_{1,t_{2}}}},\Psi$ is a smoothness penalty on edge weights, and λ is a hyperparameter selected by cross-validation (see Supplementary Information E for details).
  Once we obtain $\hat{L^{1}}$, we refine our estimate of $\bar{B^{t_{1},t_{2}}}$ using
(25) $$\hat{B^{t_{1},t_{2}}}=e^{\hat{L^{1}}\left(t_{2}-t_{1}\right)}[\hat{B^{t_{2},t_{2}}}-\left(t_{2}-t_{1}\right)1_{d\times d}].$$
3. Estimate $\bar{B^{t_{0},t_{2}}}$, and $L^{0}$ based on corresponding data and estimated $\bar{B^{t_{1},t_{2}}}$ using equation (5b). The procedure follows the same logic as in last step: we first obtain a direct sample-mean estimate of $\bar{B^{t_{0},t_{2}}}$, and then infer $L^{0}$ by solving an analogous optimization problem. Finally, we refine $\bar{B^{t_{0},t_{2}}}$ using the resulting $\hat{L^{0}}$.

**Fig. 5.**
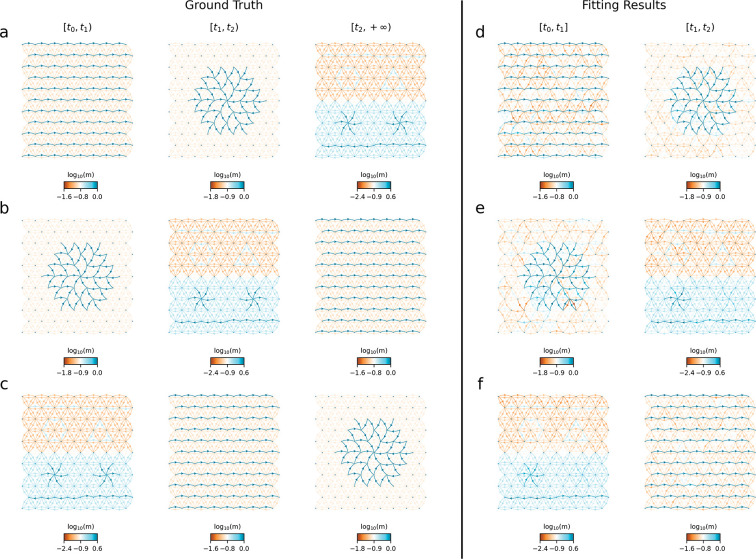
Simulation results. The first row (**a–c**) presents the ground truth topology sequences across three epochs. We define three distinct migration topologies: topology 1 is large-scale directionally migrating lineages, topology 2 is large-scale spatially converging lineages, and topology 3 is a mixutre of small scale patterns. Panel (a) shows the sequence 1→2→3, panel (b) the sequence 2→3→1, and panel (c) the sequence 3→1→2. The second row (**d–f**) shows the corresponding inferred migration patterns for each of the three settings.

The results of the simulation study are shown in Fig. 5. Figures 5(a–c) present the ground-truth topologies for each epoch. In Fig. 5a, the sequence of topologies is as follows: during epoch $\left[t_{0},t_{1}\right]$, we have a topology of large-scale directionally migrating lineages, where lineages from the left boundary migrate backward to the right boundary; during epoch $\left[t_{1},t_{2}\right]$, the topology is large-scale spatially converging lineages, where lineages converge backward to the center; and during epoch $\left[t_{2},+\infty \right)$, the topology consists of a mixture of small-scale patterns. We denote these by topology 1, topology 2, and topology 3, respectively. Thus, Fig. 5a corresponds to the topology sequence 1→2→3. Figures 5b and 5c show two other settings obtained by rotating this sequence (2→3→1 for Fig. 5b and 3→1→2 for Fig. 5c). The detailed setting and visualization of simulation parameters are provided in Supplementary Information F and Supplementary Fig. 1–3.

Figures 5(d–f) show the inferred migration patterns. In all three settings, our method largely recovers the underlying structures, including many of the small-scale patterns. The only exception is the spatially diverging lineages in topology 3, which are less clearly recovered. This might be due to fact that the center of the spatially diverging lineages is visited much less than the center of the spatially converging lineages.

In previous studies such as EEMS (Petkova et al., 2016), FEEMS (Marcus et al., 2021), and FRAME (Shen and Novembre, 2025), migration rates could only be inferred in a relative sense. Here since we directly use the data of pairwise branch lengths, we can estimate the absolute migration rates. We provide a more detailed discussion of this issue in the discussion section.

## Discussion

This study establishes an analytical foundation for pairwise genealogical processes under the structured serial coalescent. By deriving and solving evolution equations for pairwise branch length distributions and their expectations, we characterize fundamental relationships governing the interplay of migration and coalescence across time intervals. Our systematic classification identifies distinct relationship categories— represented graphically as edge classes in Figs. 2–4 —and evaluates their inference utility through parametric dependencies and computational complexity. This analysis reveals that relationships represented by vertical edges depend exclusively on migration rates, which is also validated in our analysis of relationships among length distributions of LPSC segments. This decoupling of spatial dynamics from coalescence effects provides a powerful tool to resolve the identifiability problem due to the interaction between migration and coalescence and enables fine-resolution inference of migration rates dynamics.

Leveraging this decoupling, we propose inference frameworks that fully exploits the migration-exclusive nature of relationships represented by vertical edges. Our approach employs a forward-in-time sequential inference strategy: beginning with inferring/imputing the focal quantity related to pairwise branch lengths and parameters in the oldest time point, we sequentially reconstruct migration rates (and coalescence rates) using relationships represented by vertical (and diagonal) edges. The architecture naturally accommodates time-stratified sampling and can be adapted to alternative summary statistics.

Despite these advances, several challenges remain for future work. First, the optimization and inference procedure we employ is straightforward and simple. While this may suffice for the proof-of-concept example, a more thorough investigation of the objective, penalty, and cross-validation procedures will be necessary to develop formal inference methods based on different statistics derived from pairwise branch lengths. What’s more, for expected pairwise branch length based inference, estimation accuracy depends critically on reliable estimates of expected pairwise branch lengths. In empirical applications, pairwise branch lengths are estimated from inferred genealogical trees rather than the true underlying trees. The gap between these two sources requires careful evaluation, since most existing tree inference methods assume panmixia and do not incorporate a formal migration model. In addition, imputation procedures used to address demes with no samples may introduce further errors. In a sequential inference framework based on expected pairwise branch lengths , these errors can propagate, and similar propagation issues can arise in SNP-based inference and LPSC-based inference, although the sources of error can differ. Addressing this problem will be particularly important for inference spanning many epochs. One possible strategy to reduce error propagation is a block-wise sequential approach, in which parameters are inferred jointly across multiple consecutive epochs rather than strictly one epoch at a time.

Second, our proof-of-concept example does not address the issue of long-range migration. This challenge has already been noted in previous studies (Petkova et al., 2016; Marcus et al., 2021; Shen and Novembre, 2025). Strictly local migration renders the migration-rate matrix sparse, and such sparsity can be leveraged for computational efficiency (Marcus et al., 2021) and parameter identifiability (Lundgren and Ralph, 2019). However, a strictly local network may be insufficient, as real populations may also have experienced gene flow from long-range connections. The difficulty is that these long-range sources are not known *a priori*; if they were, one could simply include them in the network. The FEEMS paper (Marcus et al., 2021) proposed two strategies to address this issue: a “greedy” algorithm akin to TreeMix (Pickrell and Pritchard, 2012) and a combination of the graphical lasso (Friedman et al., 2008) with graph Laplacian smoothing (Wang et al., 2016). The former has already been explored in FEEMSmix (Shastry et al., 2025), whereas the latter remains to be investigated.

Third, to further accelerate the inferences, it is important to adopt efficient algorithms to compute high-dimensional matrix exponentials—particularly for matrices with specific sparse structures. The case of pure migration rate estimation requires exponentiating a d×d matrix ($e^{Lt}$), while coalescence rate estimation involves a $d^{2}\times d^{2}$ matrix ($e^{St}$). While directly computing $e^{Lt}$ is still acceptable, directly computing $e^{St}$ can be computationally expensive for large d. In this case, computation and approximation strategies that maximally exploit the structure of S can be very helpful.

As the inference challenges outlined earlier are properly addressed, this framework may support powerful applications across evolutionary biology, epidemiology, and conservation. In human evolution, it can augment reconstruction of demographic history from ancient DNA, clarifying migration patterns and their connections to cultural and environmental change. In epidemiological research, it could be aid reconstructions of pathogen transmission dynamics across space and time, facilitating the monitoring of disease spread. In conservation genetics, it may help reveal how gene flow among natural populations shifts through time and how these changes are shaped by environmental pressures such as habitat loss, fragmentation, and climate change. Together, these contributions may provide new avenues to analyze population histories where spatial and temporal dimensions interact—from deep evolutionary timescales to contemporary ecological monitoring.

## Supplementary Material

Supplement 1

## Code Availability

The code to reproduce all results is available at https://github.com/ShenHaotv/When-migration-leaves-a-clean-trace.
