# Supplementary material for: When migration leaves a clean trace: Decoupling migration from coalescence in the structured serial coalescent
Source: bioRxiv. 2025 Oct 11:2025.10.10.681523. Preprint. [Version 2] doi: 10.1101/2025.10.10.681523 (PMC12632521; doi:10.1101/2025.10.10.681523)
Supplement: Supplement 1 [file media-1.pdf]

Supplementary Information for

When migration leaves a clean trace: Decoupling migration from coalescence in the Structured Serial Coalescent

Hao Shen[1] and John Novembre[1,2]

[1]Department of Human Genetics, University of Chicago
[2]Department of Ecology and Evolution, University of Chicago

Supplementary Information A:

Relationships among probability density functions of pairwise total branch length

In the main text, we have established the relationships corresponding to the vertical edges and diagonal edges, namely $(f^{t_0,t_1}, f^{t_1,t_1})$;$(f^{t_0,t_2}, f^{t_1,t_2})$; $(f^{t_1,t_2}, f^{t_2,t_2})$; $(f^{t_0,t_0}, f^{t_1,t_1})$ and $(f^{t_1,t_1}, f^{t_2,t_2})$. Here, we restate these relationships:

$$f^{t_0,t_1,b} = e^{-L^0(t_1-t_0)} f^{t_1,t_1,b-(t_1-t_0)}, \tag{S1a}$$

$$f^{t_0,t_2,b} = e^{-L^0(t_1-t_0)} f^{t_1,t_2,b-(t_1-t_0)}, \tag{S1b}$$

$$f^{t_1,t_2,b} = e^{-L^1(t_2-t_1)} f^{t_2,t_2,\, b-(t_2-t_1)}; \tag{S1c}$$

$$\mathrm{vec}(f^{t_0,b}) = \begin{cases} e^{-\frac{1}{2}S^0 b} G^0 & b \in [0, 2(t_1-t_0)) \\ e^{-S^0(t_1-t_0)}\,\mathrm{vec}(f^{t_1,b-2(t_1-t_0)}) & b \in [2(t_1-t_0), +\infty) \end{cases} \tag{S2a}$$

$$\mathrm{vec}(f^{t_1,b}) = \begin{cases} e^{-\frac{1}{2}S^1 b} G^1 & b \in [0, 2(t_2-t_1)) \\ e^{-S^1(t_2-t_1)}\,\mathrm{vec}(f^{t_2,b-2(t_2-t_1)}) & b \in [2(t_2-t_1) + \infty) \end{cases} \tag{S2b}$$

We now derive the expressions for the relationships corresponding to the horizontal edges, namely $(f^{t_0,t_0}, f^{t_0,t_1})$, $(f^{t_1,t_1}, f^{t_1,t_2})$, and $(f^{t_0,t_1}, f^{t_0,t_2})$. We begin with $(f^{t_0,t_0}, f^{t_0,t_1})$. From equation (S1a), we have

$$\mathrm{vec}(f^{t_0,t_1,b}) = \mathrm{vec}(e^{-L^0(t_1-t_0)} f^{t_1,t_1,b-(t_1-t_0)}). \tag{S3}$$

Combining this expression with (S2a), and do the reparameterization, we get the expression of $(f^{t_0,t_0}, f^{t_0,t_1})$:

$$\mathrm{vec}(f^{t_0,t_0,b}) = \begin{cases} e^{-\frac{1}{2}S^0 b} G^0 & b \in [0, 2(t_1-t_0)) \\ e^{-S^0(t_1-t_0)}\,\mathrm{vec}(e^{L^0(t_1-t_0)} f^{t_0,t_1,b-(t_1-t_0)}) & b \in [2(t_1-t_0), +\infty) \end{cases} \tag{S4}$$

Similarly, the relationship $(f^{t_1,t_1}, f^{t_1,t_2})$ can be expressed as

$$\mathrm{vec}(f^{t_1,t_1,b}) = \begin{cases} e^{-\frac{1}{2}S^1 b} G^1 & b \in [0, 2(t_2-t_1)) \\ e^{-S^1(t_2-t_1)}\,\mathrm{vec}(e^{L^1(t_2-t_1)} f^{t_1,t_2,b-(t_2-t_1)}) & b \in [2(t_2-t_1), +\infty) \end{cases} \tag{S5}$$

From equation (S1b), we also have

$$\mathrm{vec}(f^{t_0,t_2,b}) = \mathrm{vec}(e^{-L^0(t_1-t_0)} f^{t_1,t_2,b-(t_1-t_0)}). \tag{S6}$$

Combining the last two equations with equation (S1a) and after reparameterization we obtain the expression of $(f^{t_0,t_1}, f^{t_0,t_2})$:

$$
\begin{aligned}
&\operatorname{vec}(e^{L^0(t_1-t_0)} f^{t_0,t_1,b}) \\
&= \begin{cases} e^{-\frac{1}{2}S^1[b-(t_1-t_0)]} G^1 & b \in [t_1-t_0, 2t_2-t_1-t_0) \\ e^{-S^1(t_2-t_1)} \operatorname{vec}(e^{L^1(t_2-t_1)} e^{L^0(t_1-t_0)} f^{t_0,t_2,b-(t_2-t_1)}) & b \in [2t_2-t_1-t_0, +\infty) \end{cases}
\end{aligned}
\tag{S7}
$$

Supplementary Information B:

Relationships among expected pairwise total branch lengths

In the main text, we have established the relationships corresponding to the vertical edges and diagonal edges, namely $(\overline{B^{t_0,t_1}}, \overline{B^{t_1,t_1}})$; $(\overline{B^{t_0,t_2}}, \overline{B^{t_1,t_2}})$; $(\overline{B^{t_1,t_2}}, \overline{B^{t_2,t_2}})$; $(\overline{B^{t_0,t_0}}, \overline{B^{t_1,t_1}})$ and $(\overline{B^{t_1,t_1}}, \overline{B^{t_2,t_2}})$, which we restate here as:

$$\overline{B^{t_0,t_1}} = e^{-L^0(t_1-t_0)}\overline{B^{t_1,t_1}} + (t_1 - t_0)1_{d\times d}, \tag{S8a}$$

$$\overline{B^{t_0,t_2}} = e^{-L^0(t_1-t_0)}\overline{B^{t_1,t_2}} + (t_1 - t_0)1_{d\times d}, \tag{S8b}$$

$$\overline{B^{t_1,t_2}} = e^{-L^1(t_2-t_1)}\overline{B^{t_2,t_2}} + (t_2 - t_1)1_{d\times d}; \tag{S8c}$$

$$\mathrm{vec}(\overline{B^{t_0}} - \overline{B^{0*}}) = e^{-S^0(t_1-t_0)}\,\mathrm{vec}(\overline{B^{t_1}} - \overline{B^{0*}}), \tag{S9a}$$

$$\mathrm{vec}(\overline{B^{t_1}} - \overline{B^{1*}}) = e^{-S^1(t_2-t_1)}\,\mathrm{vec}(\overline{B^{t_2}} - \overline{B^{1*}}). \tag{S9b}$$

Here we would like to derive the expression of $(\overline{B^{t_0,t_0}}, \overline{B^{t_0,t_1}})$; $(\overline{B^{t_1,t_1}}, \overline{B^{t_1,t_2}})$; $(\overline{B^{t_0,t_1}}, \overline{B^{t_0,t_2}})$, which correspond to the horizontal edges. We first study $(\overline{B^{t_0,t_0}}, \overline{B^{t_0,t_1}})$. From equation (S8a), we have

$$\mathrm{vec}(\overline{B^{t_0,t_1}}) = \mathrm{vec}(e^{-L^0(t_1-t_0)}\overline{B^{t_1,t_1}} + (t_1 - t_0)1_{d\times d}), \tag{S10}$$

and from equation (S11),

$$\mathrm{vec}(\overline{B^{t_0,t_0}} - \overline{B^{0*}}) = e^{-S^0(t_1-t_0)}\,\mathrm{vec}(\overline{B^{t_1,t_1}} - \overline{B^{0*}}). \tag{S11}$$

Combining the two gives the expression of $(\overline{B^{t_0,t_0}}, \overline{B^{t_0,t_1}})$:

$$\mathrm{vec}(\overline{B^{t_0,t_0}} - \overline{B^{0*}}) = e^{-S^0(t_1-t_0)}\,\mathrm{vec}(e^{L^0(t_1-t_0)}\overline{B^{t_0,t_1}} - (t_1 - t_0)1_{d\times d} - \overline{B^{0*}}). \tag{S12}$$

In a similar way, the relationship $(\overline{B^{t_0,t_0}}, \overline{B^{t_1,t_1}})$ can be written as

$$\mathrm{vec}(\overline{B^{t_1,t_1}} - \overline{B^{1*}}) = e^{-S^1(t_2-t_1)}\,\mathrm{vec}(e^{L^1(t_2-t_1)}\overline{B^{t_0,t_1}} - (t_2 - t_1)1_{d\times d} - \overline{B^{1*}}). \tag{S13}$$

Finally, from equation (S8b), we obtain

$$\mathrm{vec}(\overline{B^{t_0,t_2}}) = \mathrm{vec}(e^{-L^0(t_1-t_0)}\overline{B^{t_1,t_2}} + (t_1 - t_0)1_{d\times d}). \tag{S14}$$

Combining this with equations (S11) and (S13), we derive the relationship $(\overline{B^{t_1,t_1}}, \overline{B^{t_2,t_2}})$:

$$\begin{aligned}&\mathrm{vec}(e^{L^0(t_1-t_0)}\overline{B^{t_0,t_1}} - (t_1 - t_0)1_{d\times d} - \overline{B^{1*}})\\ =&e^{-S^1(t_2-t_1)}\,\mathrm{vec}(e^{L^1(t_2-t_1)}e^{L^0(t_1-t_0)}\overline{B^{t_0,t_2}} - (t_2 - t_0)1_{d\times d} - \overline{B^{1*}}).\end{aligned} \tag{S15}$$

Supplementary Information C:

Relationships among survival functions of LPSC segment lengths

Here we give a detailed derivation of relationships among survival functions of LPSC segment lengths given in the main text. From main text we have

$$\rho^{x,y,\mu} = \int_0^{+\infty} e^{-rb\mu} f^{x,y,b} db. \tag{S16}$$

Writing $L^0 + r\mu I, L^1 + r\mu I$ as L^0_μ, L^1_μ and using equation (S1a) yields

$$\begin{aligned} \rho^{t_0,t_1,\mu} &= \int_0^{+\infty} e^{-rb\mu} f^{x,y,b} db \\ &= e^{-L^0(t_1-t_0)} \int_0^{+\infty} e^{-rb\mu} f^{t_1,t_1,b-(t_1-t_0)} db \\ &= e^{-L^0_\mu(t_1-t_0)} \rho^{t_1,t_1,\mu} \end{aligned} \tag{S17}$$

Applying equation (S1b) and (S1c) similarly gives

$$\rho^{t_0,t_2,\mu} = e^{-L^0_\mu(t_1-t_0)} \rho^{t_1,t_2,\mu} \tag{S18}$$

and

$$\rho^{t_1,t_2,\mu} = e^{-L^1_\mu(t_2-t_1)} \rho^{t_2,t_2,\mu}. \tag{S19}$$

The three equations above show the relationships represented by the vertical edges, namely $(\rho^{t_0,t_1,\mu}, \rho^{t_1,t_1,\mu})$, $(\rho^{t_0,t_2,\mu}, \rho^{t_1,t_2,\mu})$, and $(\rho^{t_1,t_2,\mu}, \rho^{t_2,t_2,\mu})$. We can perform a similar analysis for the relationships represented by the diagonal edges, i.e. $(\rho^{t_0,t_0,\mu}, \rho^{t_1,t_1,\mu})$ and $(\rho^{t_1,t_1,\mu}, \rho^{t_2,t_2,\mu})$. Let $\rho^{x,x,\mu} = \rho^{x,\mu}$ and $S^0 + 2r\mu I = S^0_\mu$. Using equations (S2a), we obtain

$$\begin{aligned} \mathrm{vec}(\rho^{t_0,\mu}) &= \int_0^{2(t_1-t_0)} e^{-rb\mu} e^{-\frac{1}{2}S^0 b} G^0 db \\ &+ \int_{2(t_1-t_0)}^{+\infty} e^{-rb\mu} e^{-S^0(t_1-t_0)} \mathrm{vec}(f^{t_1,b-2(t_1-t_0)}) db \\ &= 2[I - e^{-S^0_\mu(t_1-t_0)}](S^0_\mu)^{-1} G^0 + e^{-S^0_\mu(t_1-t_0)} \mathrm{vec}(\rho^{t_1,\mu}). \end{aligned} \tag{S20}$$

Similarly, let $S^1 + 2r\mu I = S^1_\mu$, and using equation (S2b) we have

$$\mathrm{vec}(\rho^{t_1,\mu}) = 2[I - e^{-S^1_\mu(t_2-t_1)}](S^1_\mu)^{-1} G^1 + e^{-S^1_\mu(t_2-t_1)} \mathrm{vec}(\rho^{t_2,\mu}). \tag{S21}$$

To get the expression of the relationship $(\rho^{t_0,t_0,\mu}, \rho^{t_0,t_1,\mu})$, we rewrite equation (S20) as

$$\mathrm{vec}(\rho^{t_0,t_0,\mu}) = 2[I - e^{-S^0_\mu(t_1-t_0)}](S^0_\mu)^{-1} G^0 + e^{-S^0_\mu(t_1-t_0)} \mathrm{vec}(\rho^{t_1,t_1,\mu}). \tag{S22}$$

Combine this equation with equation (S17), we obtain

$$\mathrm{vec}(\rho^{t_0,t_0,\mu}) = 2[I - e^{-S_\mu^0(t_1-t_0)}](S_\mu^0)^{-1}G^0 + e^{-S_\mu^0(t_1-t_0)}\,\mathrm{vec}(e^{L_\mu^0(t_1-t_0)}\rho^{t_0,t_1,\mu}). \tag{S23}$$

Similarly, the relationship $(\rho^{t_1,t_1,\mu}, \rho^{t_1,t_2,\mu})$ can be expressed as

$$\mathrm{vec}(\rho^{t_1,t_1,\mu}) = 2[I - e^{-S_\mu^1(t_2-t_1)}](S_\mu^1)^{-1}G^1 + e^{-S_\mu^1(t_2-t_1)}\,\mathrm{vec}(e^{L_\mu^1(t_2-t_1)}\rho^{t_1,t_2,\mu}). \tag{S24}$$

Combing equation (S24) with equation (S17) and (S18), we get the expression of $(\rho^{t_0,t_1,\mu}, \rho^{t_0,t_2,\mu})$:

$$\begin{aligned} &e^{L_\mu^0(t_1-t_0)}\,\mathrm{vec}(\rho^{t_0,t_1,\mu}) \\ =&2[I - e^{-S_\mu^1(t_2-t_1)}](S_\mu^1)^{-1}G^1 + e^{-S_\mu^1(t_2-t_1)}\,\mathrm{vec}(e^{L_\mu^1(t_2-t_1)}e^{L_\mu^0(t_1-t_0)}\rho^{t_0,t_2,\mu}) \end{aligned} \tag{S25}$$

Supplementary Information D:

Expected pairwise total branch length and sample covariance structure under the (structured) serial coalescent

Here we establish the relationship between the expected pairwise total branch lengths and sample covariance structure under the (structured) serial coalescent. This connection can be established through analysis similar to McVean (2009). For a given snp, let the allele state of haploid sample X and Y be Z_X and Z_Y, both could have value 0 or 1, Conditioned on the event S that only 1 mutation happens in the geneology (which includes sample X, Y and other samples), and take the limit as the mutation rate θ approaches zero, the expectation of Z_X can be expressed as

$$E(Z_X|S) = \frac{\Pr(Z_X = 1, S)}{P(S)} = \lim_{\theta \to 0} \frac{E(\theta(t_G^{MRCA} - t_X)e^{-\theta B_{tot}})}{E(\theta B_{tot} e^{-\theta B_{tot}})} = \frac{\overline{t_G^{MRCA}} - t_X}{\overline{B_{tot}}}, \quad \text{(S26)}$$

where $\overline{t_G^{MRCA}}$ is the expected time of the most recent common ancestor of the geneology (i.e. all samples) , $\overline{B_{tot}}$ is the expected total branch length of the geneology. Similarly, Z_Y annd $Z_X Z_Y$ can be expressed as

$$E(Z_Y|S) = \frac{\overline{t_G^{MRCA}} - t_Y}{\overline{B_{tot}}}, \quad \text{(S27a)}$$

$$E(Z_X Z_Y|S) = \frac{\overline{t_G^{MRCA}} - \overline{t_{XY}^{MRCA}}}{\overline{B_{tot}}} = \frac{2\overline{t_G^{MRCA}} - (t_X + t_Y) - \overline{B_{XY}}}{2\overline{B_{tot}}}, \quad \text{(S27b)}$$

where $\overline{B_{XY}}$ is the expected pairwise total branch length between X and Y. Here t_X and t_Y are known parameters, thus to establish the connection between $E(Z_X Z_Y|S)$ and $\overline{B_{XY}}$, we only need to properly address the remaining constant $\overline{t_G^{MRCA}}$ and $\overline{B_{tot}}$. From the practice of EEMS (Petkova et al., 2016), FEEMS (Marcus et al., 2021) and FRAME (Shen and Novembre, 2025), we know $\overline{t_G^{MRCA}}$ can be eliminated by applying a contrast, and $\overline{B_{tot}}$ can be treated as a nuisance parameter.

Supplementary Information E:

Objective function and cross validation

Here we provide a detailed introduction to the optimization procedure used in the simulation section of the main text.

The lineage migration process in each epoch can be represented by a directed weighted graph. We number the nodes from 1 to d, and use the ordered pair (i,j) to represent an edge from node i to node j. Let Ω denote the edge set under this representation, and define

$$\Omega_i = \{(i,t) \in \Omega\} \cup \{(s,i) \in \Omega\},$$

the set of edges connected to node i. We assume that Ω and Ω_i remain fixed across epochs, while only the edge weights vary.

Suppose we have $\widehat{B^{t_1,t_2}_{\text{sample}}}$ and $\widehat{B^{t_2,t_2}}$ available, and would like to infer L^1 using the equation

$$\overline{B^{t_1,t_2}} - (t_2 - t_1)\, 1_{d\times d} = e^{-L^1(t_2-t_1)}\overline{B^{t_2,t_2}}. \tag{S28}$$

In the main text, we assume that samples are available from all demes. For empirical datasets, however, some demes may lack samples, and the same issue also arises in cross-validation. To handle this, suppose we observe o^1 demes at time t_1 with indices $O^1 = \{\alpha^1_1, \ldots, \alpha^1_{o^1}\}$, and o^2 demes at time t_2 with indices $O^2 = \{\alpha^2_1, \ldots, \alpha^2_{o^2}\}$. Then $\widehat{B^{t_1,t_2}_{\text{sample}}}$ is an $o^1 \times o^2$ matrix.

We define selection matrices $C^1 \in \mathbb{R}^{o^1\times d}$ and $C^2 \in \mathbb{R}^{o^2\times d}$, where $C^1_{ij} = 1$ if $j = \alpha^1_i$ (and 0 otherwise), and $C^2_{ij} = 1$ if $j = \alpha^2_i$ (and 0 otherwise). Equation (S28) then becomes

$$C^1\left[\overline{B^{t_1,t_2}} - (t_2 - t_1)\, 1_{d\times d}\right](C^2)^T = C^1\left[e^{-L^1(t_2-t_1)}\overline{B^{t_2,t_2}}\right](C^2)^T. \tag{S29}$$

The data we have at hand corresponding to left hand-side of the equation is $\widehat{B^{t_1,t_2}_{\text{sample}}} - (t_2 - t_1)1_{o^1\times o^2}$, which we denote by A_1; The data corresponding to the right hand-side of the equation is $C^1[e^{-L^1(t_2-t_1)}\widehat{B^{t_2,t_2}}](C^2)^T$, which we denote by A_2, we can then define a matrix of relative error $\varepsilon^1_r \in R^{o^1\times o^2}$, such that

$$(\varepsilon^1_r)_{ij} = \frac{|(A_2 - A_1)_{ij}|}{|(A_1)_{ij}|}. \tag{S30}$$

The optimization problem can then be written as

$$\widehat{L^1} = \underset{L^1\in\mathcal{D}}{\operatorname{argmin}}\,(\|\ \varepsilon^1_r\ \|^2 + \lambda\Psi), \tag{S31}$$

where $\mathcal{D}$ is the region that all edges have positive weight; Ψ is a smoothness penalty defined by

$$\Psi = \frac{1}{2}\sum_{i=1}^{d}\sum_{\substack{(i_1,j_1)\in\Omega_i,\\(i_2,j_2)\in\Omega_i}}\frac{1}{|\Omega_i|^2}[\log(m_{i_1j_1}) - \log(m_{i_2j_2})]^2, \tag{S32}$$

and λ is a hyperparameter controlling the strength of regularization.

For each fixed λ, we solve the optimization problem using L-BFGS (Byrd et al., 1995), with gradients computed automatically via PyTorch (Paszke et al., 2019). The optimal λ is then selected through cross-validation.

Specifically, for a chosen k, we partition the observed demes at time t_1 into k folds, $O_1^1, \ldots, O_k^1$, and the observed demes at t_2 into $O_1^2, \ldots, O_k^2$. For each i, we hold out (O_i^1, O_i^2) and train on the remaining demes. Let $\widehat{L_{\text{train}}^1}$ be the estimate obtained from the training set. In this case, only entries of equation (S28) with row indices in $O_{\text{train}}^1 = O^1 \setminus O_i^1$ and column indices in $O_{\text{train}}^2 = O^2 \setminus O_i^2$ are used for inference. The held-out entries—those with row indices in O_i^1 or column indices in O_i^2—serve as the test set.

The validation error for fold i is then defined as square summation of the relative errors of these entries. Or in an equivalent format, let $(\varepsilon_r^1)_{\text{train}}$ be a matrix computed from $A_1 = \widehat{B_{\text{train}}^{t_1,t_2}} - (t_2 - t_1)1_{|O_{\text{train}}^1| \times |O_{\text{train}}^2|}$ and $A_2 = C_{\text{train}}^1[e^{-L^1(t_2-t_1)}\widehat{B^{t_2,t_2}}](C_{\text{train}}^2)^T$, where $\widehat{B_{\text{train}}^{t_1,t_2}}, C_{\text{train}}^1, C_{\text{train}}^2$ represents the expected pairwise total branch length matrix and selection matrix for training demes, the validation error can be defined as $\| \varepsilon_r^1 \|^2 - \| (\varepsilon_r^1)_{\text{train}} \|^2$.

Supplementary Information F:

Simulation setting

We use `msprime` (Baumdicker et al., 2022) to simulate the structured serial coalescent process. We define three time points, $t_0 = 0$, $t_1 = 1$, and $t_2 = 2$, which partition the process into three epochs: $[t_0, t_1)$, $[t_1, t_2)$, and $[t_2, t_\infty)$. In each epoch, one of three topologies is applied: large-scale directionally migrating lineages, large-scale spatially converging lineages, or a mixture of small-scale patterns (as shown in Fig. 5 of the main text).

In both the large-scale directionally migrating lineages and large-scale spatially converging lineages, the base migration rate (corresponding to the orange edges) is set to 0.1, while the blue edges have migration rates 1, which are ten times higher. In the topology combining small-scale patterns, the migration rates along the middle row are 0.1. The upper half of the graph have a base migration rate of 0.03, shown as orange edges. The lower half of the graph have a base migration rate of 0.3, shown as blue edges. All patterns exhibit migration rates that are 10 times higher than the surrounding base migration rates. Specifically, the patterns in the upper half of the graph will have a migration rate of 0.3, while patterns in the lower half will have migration rates of 3.

Effective population sizes are chosen at random by sampling log(Ne) uniformly from the interval $[-1, 1]$. The resulting values used in the simulations are visualized in Supplementary Figs. 1–3.

From each deme, 10 lineages are sampled, and the structured serial coalescent simulation is performed with the specified migration and coalescence rates in each epoch. Each simulation yields a tree sequence. We repeat this procedure 10000 times and conduct inference using the pairwise total branch lengths computed from the resulting tree sequences.

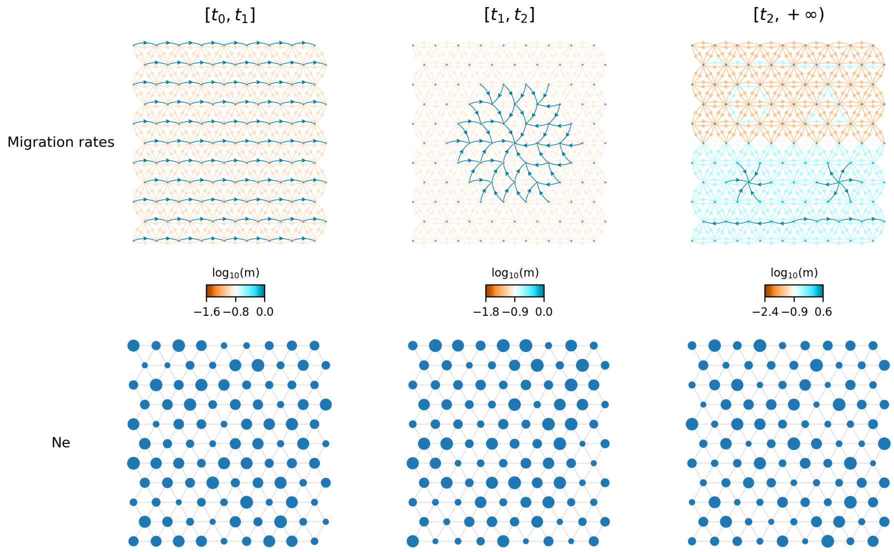

Fig. S1. Migration rates and effective population size settings for the topology sequence in Fig. 5a of the main text

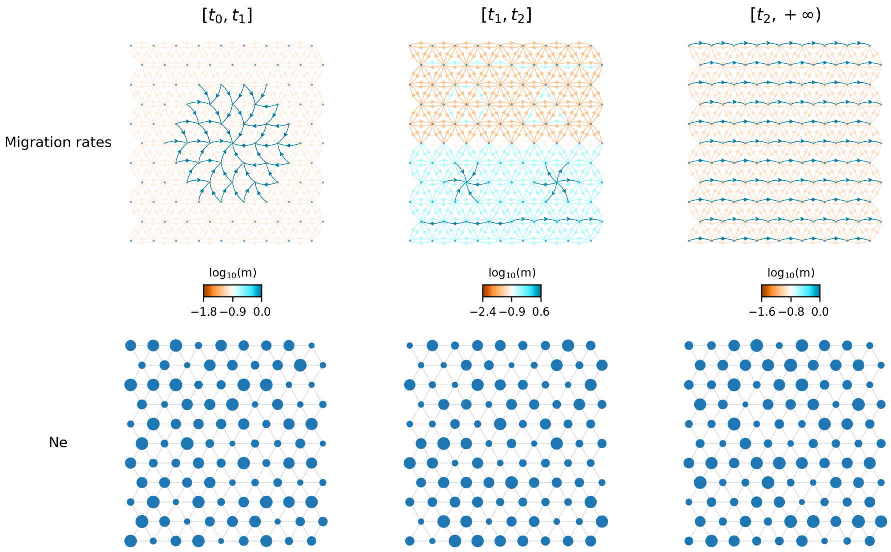

Fig. S2. Migration rates and effective population size settings for the topology sequence in Fig. 5b of the main text

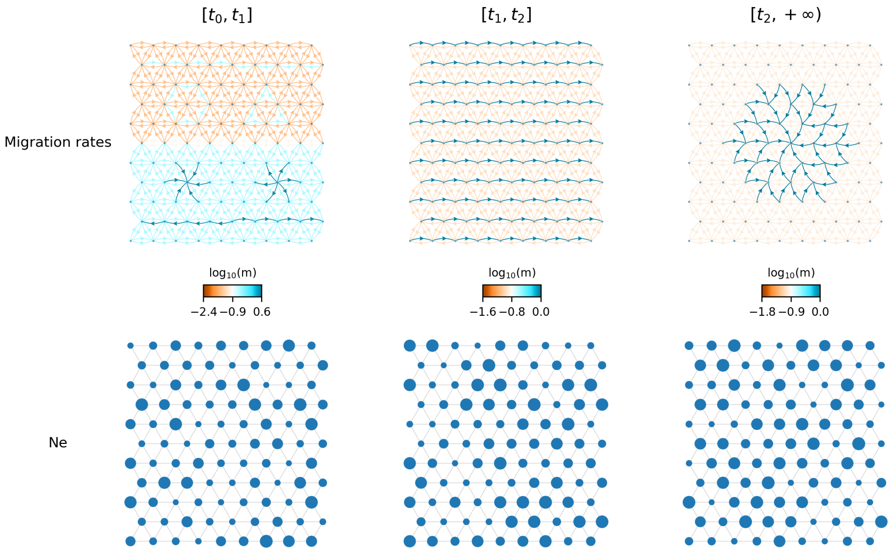

Fig. S3. Migration rates and effective population size settings for the topology sequence in Fig. 5c of the main text

References

Baumdicker, F. et al. (2022). Efficient ancestry and mutation simulation with msprime 1.0. *Genetics*, 220:iyab229.

Byrd, R., Lu, P., Nocedal, J., and Zhu, C. (1995). A limited memory algorithm for bound constrained optimization. *SIAM J. Sci. Comput.*, 16(5):1190–1208.

Marcus, J., Ha, W., Barber, R. F., and Novembre, J. (2021). Fast and flexible estimation of effective migration surfaces. *eLife*, 10:e61927.

McVean, G. (2009). A genealogical interpretation of principal components analysis. *PLoS Genetics*, 5:e1000686.

Paszke et al. (2019). Pytorch: An imperative style, high-performance deep learning library. arXiv preprint arXiv:1912.01703. `https://arxiv.org/abs/1912.01703`.

Petkova, D., Novembre, J., and Stephens, M. (2016). Visualizing spatial population structure with estimated effective migration surfaces. *Nature Genetics*, 48:94–100.

Shen, H. and Novembre, J. (2025). Fine-resolution asymmetric migration estimation. bioRxiv. `https://doi.org/10.1101/2025.05.29.656894`.
